# Evaluation of the Regional Reference Centre on Drugs of Juiz de Fora, Brazil

**DOI:** 10.1186/1940-0640-8-S1-A64

**Published:** 2013-09-04

**Authors:** Pollyanna Santos da Silveira, Pedro Henrique Antunes da Costa, Leonardo Fernandes Martins, Telmo Mota Ronzani

**Affiliations:** 1 Federal University of Juiz de Fora, Brazil, Center for Research, Intervention and Evaluation for Alcohol and Drugs (CREPEIA), Juiz de Fora, Brazil

## 

There are some evidences in Brazil and others countries pointing out insufficient resources and actions to promote prevention and rehabilitation for substance abuse related disorders. Also the lack of professional qualification contributes to this scenario, generating knowledge deficiencies and inadequate practices. The study evaluates the Regional Reference Centre on Drugs of Juiz de Fora, Brazil (CRR-JF). The CRR-JF aims to train professionals of the local health sector (NHS) and social care system increasing their knowledge of screening and brief intervention (SBI) practices on the use of drugs and strengthening health care activities. Five courses are provided: Motivational Counseling and Brief Intervention, Case Management and Social Reintegration of Drug Users, Improvement of Medical Practices about Drugs, and Comprehensive Drug Care for Hospital’s Professionals. Questionnaires were applied measuring participant satisfaction and changes in the practices. The added workload of the courses was 360 hours. A total of 180 professionals attended the courses (community health workers, social workers, nurses, doctors, psychologists and others). According to results, 83.2% of participants were satisfied or very satisfied about the courses attended; 61.3% evaluated the content of the courses as good, 28% as great, and 60% were very satisfied or satisfied with the applicability of the course content. Regarding SBI, the participants showed a significant increase in their level of trust and ability to approach users of psychoactive substances and counsel them to reduce or stop their consumption. Besides providing knowledge acquisition, participation in the CRR-JF enabled greater discussion of the practices carried out, reflecting on the work of professionals in the development of more effective interventions in prevention, promotion, and psychosocial rehabilitation of users of psychoactive substances. Acknowledgments: National Secretary of Policy on Drugs (SENAD), Coordination for the Improvement of Higher Education Personnel (CAPES).

